# Essays on Infant Therapeutics

**Published:** 1852-01

**Authors:** 


Art. XII.—*Essays on Infant Therapeutics.* To which are added, observations on Ergot; history of the origin of the use of mercury in inflammatory complaints; together with the statistics of the deaths from poisoning in New York, in the years 1841—'2—'3. By John B. Beck, M. D., Professor of Materia Medica and Medical Jurisprudence in the College of Physicians and Surgeons of the University of New York; &c. &c. Second edition, enlarged and revised. New York. Wm. E. Dean. 1852.

The preparation of a new edition of this admirable series of essays occupied the closing hours of the life of the eminent physician and teacher, Professor J. B. Beck.—Few ever surpassed him in devotion to his profession.—He zealously labored for its advancement, and contributed largely to its success.

The essays before us are worthy of their admirable author. We are not prepared to subscribe fully to all the opinions and inferences of the work, but even in our differences of opinion, we cheerfully bear testimony to the calm, elevated and practical philosophy of the author, and there are few practitioners of medicine who will fail to derive information from a study of the subjects of this book.

The following is a list of the essays:

1. "On the effects of opium on the young subject."
2. "On the effects of emetics on the young subject."
3. "On the effects of mercury on the young subject."
4. "On the effects of blisters on the young subject—sinapisms."
5. "On the effects of blood-letting on the young subject."
6. "On ergot."
7. "Origin of the use of mercury in inflammatory complaints."
8. "On the deaths from poisoning in New York, &c."

An appendix is added, and contains some curious and useful matters. They consist of the following subjects:

1. "Death of a child from one drop of laudanum."
2. "Trial of a nurse for poisoning a child by giving two drops of laudanum."
3. "Case where a large quantity of opium was given to a child for strangulated hernia"
4. "On the sedative effects of tartar emetic."
5. "Death of a child from the use of tartar emetic."
6. "On bleeding *ad deliquium* in croup."
7. "On ergot, by Dr. Beatty, Dublin."
8. "On ergot, by Dr. Hardy, of Dublin."
9. "On ergot," from the *Charleston Medical Journal and Review*.

These subjects offered a fine scope to the well trained mind of Professor Beck, and he treated them with a full sense of their importance, and of his responsibilities to his profession, and to humanity. On a great variety of points, our limited experience corroborates the views of the author ; on others w eare not prepared for full corroboration. But we earnestly commend this little work to the attention of readers of medical books.

The book is filled with a variety of useful instruction, on points of vital importance to the medical profession.

